# *P4HB*, a Novel Hypoxia Target Gene Related to Gastric Cancer Invasion and Metastasis

**DOI:** 10.1155/2019/9749751

**Published:** 2019-07-30

**Authors:** Jun Zhang, Shuai Guo, Yue Wu, Zhi-chao Zheng, Yue Wang, Yan Zhao

**Affiliations:** ^1^Gastric Cancer Department, Liaoning Province Cancer Hospital & Institute (Cancer Hospital of China Medical University), No. 44 Xiaoheyan Road, Dadong District, Shenyang, Liaoning, 110042, China; ^2^Emergency Department, Shengjing Hospital of China Medical University, 36 Sanhao St., Heping District, Shenyang, Liaoning, 110003, China

## Abstract

Gastric cancer (GC) is a common tumor-associated lethal disease, and invasiveness and metastasis are primary challenges in its clinical treatment. Hypoxia microenvironment cannot be ignored in the process of metastasis. Hypoxia inducible factor-1*α* (HIF-1*α*) is the core component of the hypoxia signaling pathway. The aim of this study was to identify potential hub genes and signaling pathways associated with HIF-1*α*. We explored the invasiveness- and metastasis-associated phenotype of GC via bioinformatics analysis and molecular studies. Differentially expressed genes (DEGs) were identified in GC cells and HIF-1*α*-knockdown GC cells. Gene ontology (GO) and Kyoto Encyclopedia of Genes and Genomes (KEGG) pathway enrichment analyses were performed, and a protein-protein interaction (PPI) network was constructed. Hub genes were identified via centrality analysis and Molecular Complex Detection (MCODE) module analysis. The findings suggested that prolyl 4-hydroxylase beta polypeptide (*P4HB*) has strong associations with HIF-1*α*. Further, we observed that HIF-1*α* and* P4HB* were upregulated in SGC-7901 and BGC-823 cells. In addition, inhibition of HIF-1*α* expression reduced invasion and metastasis in GC cells; this effect was partially reversed by* P4HB* overexpression. Our results confirm that* P4HB* plays a significant role in the regulatory network of HIF-1*α*. Therefore, HIF-1*α* and* P4HB* may be considered potential biomarkers of GC.

## 1. Introduction

Gastric cancer (GC) is the most common malignant tumor of the digestive system. Although progress has been reported in terms of treatment, it is still the second leading cause of cancer-related death [1]. Early diagnosis and control of invasion and metastasis are important research directions [2]. Therefore, the identification of biomarkers and their underlying molecular mechanisms in GC is critical. Evidence shows that the tumor hypoxic microenvironment is attributed to tumor progression and metastasis [3]. Hypoxia induction factors (HIFs) play a synergistic role in regulating the response of cells to hypoxia [4]. In the hypoxic region of a solid tumor, these factors accelerate cell dissemination, promote angiogenesis, and advance cancer cells to a metastatic phenotype [5]. In particular,* HIF-1α *upregulates epithelial–mesenchymal transition- (EMT-) related transcription factors and is closely related to the prognosis of GC [6, 7]. However, the molecular mechanism of hypoxia affecting GC metastasis has not been fully elucidated.

Biochips and next-generation sequencing (NGS) have been used extensively to analyze gene expression in medical oncology. Recent gene expression profiling studies of GC in the last decade have revealed differentially expressed genes (DEGs) related to different signaling pathways. Based on these results, a plethora of molecular targeted drugs have been approved in the last ten years and used in clinical practice. However, the efficacy of molecular targeted drugs has not met expectations, and the anticipated huge breakthrough in treatment resulting from their use has not been achieved [8]. Comparative analysis of the DEGs identified by various studies has revealed relatively limited repeatability, and a biomarker of invasion and metastasis in GC has not yet been identified. It is becoming clear that the identification of singular DEGs is not a sufficient basis for making therapeutic drugs [9]; more complex and sophisticated software tools and algorithms are necessary. Proteogenomic analysis provides a functional context to annotate genomic abnormalities with carcinogenesis and prognostic value [10]. Cai YD et al. [11] have proved the effectiveness of the protein-protein interaction (PPI) network in predicting breast cancer metastasis. Rohwer et al. [12] collected cell samples and examined DEGs in* HIF-1α*-deficient gastric cancer AGS cells (KD-AGS) and scrambled gastric cancer AGS cells (SCR-AGS) [12]. Elucidation of the interactions between DEGs, particularly as they relate to the protein-protein interaction (PPI) network, is critical.

In this study, raw data (GSE57200) were downloaded from the Gene Expression Omnibus (GEO, http://www.ncbi.nlm.nih.gov/geo/), which allows users to search and download gene expression profiles from various experiments. Package edgeR was applied to identify DEGs in the gene expression profiles of KD and SCR. Gene ontology (GO) [13, 14], Kyoto Encyclopedia of Genes and Genomes (KEGG) [15], and PPI network analysis were used to study and identify changes in pathways and hub genes.

Prolyl 4-hydroxylase beta polypeptide (*P4HB*), one of the hub genes in the beta subunit of prolyl 4-hydroxylase, belongs to the protein disulfide isomerase (PDI) family.* P4HB* is a highly abundant multifunctional enzyme which acts as an endoplasmic reticulum (ER) chaperone to inhibit the aggregation of misfolded proteins [16]. In previous studies, we confirmed* P4HB *was overexpressed in GC tissue and acted as a potential prognostic marker of GC [7]. Besides,* P4HB* was also overexpressed in hepatocellular carcinoma [17, 18] and non-small-cell lung cancer [19]. Higher* P4HB* expression is closely linked with drug resistance in malignant glioma [20]. Further, high levels of PDIs are associated with lymphatic metastases of cancers, as confirmed by proteomic and protein functional studies [21]. These studies confirm the potential carcinogenicity of* P4HB*. However, the role of* P4HB* in invasion and metastasis of GC and its relationship with* HIF-1α* are still unclear.

In this study, we examined* P4HB* and* HIF-1α* expression in GC cells and gastric tissue samples. We also evaluated the interactions between* P4HB* and* HIF-1α* in the GC invasion and metastasis phenotype. The present findings indicated that* HIF-1α* suppresses the expression of* P4HB* and promotes GC invasion and metastasis.

## 2. Materials and Methods

### 2.1. Tissues and Ethical Statement

Forty paired GC and nontumorous adjacent tissues were obtained from patients at the Liaoning Province Cancer Hospital & Institute between 2015 and 2017. Meanwhile, this study was approved by the institutional review board of the Liaoning Province Cancer Hospital & Institute. Informed consent was obtained from patients before surgery.

### 2.2. Biochip Data and Identification of DEGs

The original GSE57200 data were downloaded from the GEO database. GSE57200, which was tested using the GPL6883 (HumanRef-8 v3.0 Gene Expression BeadChip, Illumina, San Diego, CA) and GPL10558 (HumanHT-12 v4.0 Expression BeadChip, Illumina) platforms, was submitted by Rohwer et al. In this article, we mainly discuss the data based on the GPL10558 platform. The GSE57200 (GPL10558 platform) dataset contained five samples, including three* HIF-1α*-deficient gastric cancer AGS cells established by transduction of lentiviral shRNA and two SCR gastric cancer AGS cells as control. Package edgeR was applied to identify DEGs. ggplot2 was used to depict the heatmap and volcano plot. Hierarchical clustering analysis was performed to categorize the data into two groups with similar expression in KD-AGS and SCR-AGS.

### 2.3. Gene Ontology and Pathway Enrichment Analysis of DEGs

DEG data were uploaded to Database for Annotation, Visualization and Integrated Discovery (DAVID) version 6.8 (https://david.ncifcrf.gov/) [22] to obtain categories as follows: biological process FAT, cell component FAT, and molecular function FAT datasets of GO and KEGG pathway enrichment analysis.* P *< 0.05 was considered statistically significant.

### 2.4. Integration of Protein-Protein Interaction Network and Module Analysis

Genes form networks based on their interactions [23]. The Search Tool for the Retrieval of Interacting Genes (STRING, https://string-db.org/) database is an online tool designed to evaluate PPI network data. We uploaded the DEGs data to STRING to evaluate the interactions between DEGs; scores of experimentally validated interactions > 0.4 were treated as significant. We used the Cytoscape 3.5.0 software to visualize the results of PPI networks [24]. Further, Cytoscape was used to perform centrality analysis according to local (degree and clustering coefficient) and global (betweenness, closeness, and stress) scales. The top 10 genes in the 5 types of centralities were chosen as candidates. Genes appearing more than twice were defined as hub genes. The Molecular Complex Detection (MCODE) plug-in was used to filter and analyze the PPI network module in Cytoscape. MCODE scores > 3 combined with a number of nodes > 4 were selected as significant.

### 2.5. The Kaplan-Meier Plotter

The Kaplan-Meier plotter (www.kmplot.com) database was used to evaluate the prognostic significance of the mRNA expression of* P4HB* in GC. The database included gene expression and clinical data for lung cancer, ovarian cancer, gastric cancer, and breast cancer. According to the median expression of* P4HB* (high vs. low expression), patient samples were divided into two groups to assess the prognostic value of* P4HB*. The Kaplan-Meier survival plot was used to analyze the overall survival of patients with GC.* P *< 0.05 was considered to represent statistical significance.

### 2.6. Cell Culture

GSE-1 (human normal gastric epithelial cell line) and SGC-7901 and BGC-823 (human gastric cancer cell lines) were obtained from China Medical University (Shenyang, China). Cells were cultured in RPMI 1640 medium supplemented with 10% fetal bovine serum (FBS, Invitrogen, Carlsbad, CA, USA), 100 U/mL penicillin, and 100 *μ*g/mL streptomycin (Invitrogen) at 37°C under 5% CO_2_ and 1% O_2_. All experiments were repeated three times independently.

### 2.7. RNA Extraction and Real-Time Reverse Transcription-Polymerase Chain Reaction (RT-PCR)

Total RNAs from cells and tissue were isolated with the TRIzol (Invitrogen) cell separation reagent according to the manufacturer's instructions. The Promega cDNA core kit (Promega, Madison, WI, USA) used to generate complementary DNA from 500 ng of total RNA. SYBR Master Mixture (Takara Bio, Inc., Kusatsu, Japan) was used for real-time PCR (LightCycler 480, Roche AG, Basel, Switzerland). Each sample was analyzed in three times.* U6* worked as loading control. Fold changes of mRNA expression in different cells were determined by 2^-△△CT^ normalization. The following primers were used:* HIF-1α*, forward 5′- GATCACCCTCTTCGTCGCTT -3′ and reverse 5′- AAAGGCAAGTCCAGAGGTGG -3′;* P4HB*, forward 5′- GGAATGGAGACACGGCTTC -3′ and reverse 5′- TTCAGCCAGTTCACGATGTC-3′.

### 2.8. Western Blot Analysis

10–15% SDS polyacrylamide gel and PVDF membranes were used to separate and transfer protein (20 *μ*g) from cells. The TBS-Tween buffer (20 mM Tris-HCl, 5% nonfat milk, 150 mM NaCl, and 0.05% Tween-20, pH 7.5) was used to block membranes for 1 hour at 21°C after blotting. Then the members were incubated with primary antibodies overnight at 4°C (HIF-1*α*, 1:400, Abcam, Cambridge, UK;* P4HB*, 1:200, Boster Biological Technology, Pleasanton, CA, USA; and *β*-actin, 1:4,000, Santa Cruz Biotechnology, Santa Cruz, CA, USA). Finally, members were incubated with a secondary antibody (1:5,000, Santa Cruz Biotechnology). *β*-actin acted as a control. The grey value of proteins was measured by ImageJ (NIH, Bethesda, MD, USA). Averages of three independent experiments were presented as the final data.

### 2.9. Immunohistochemistry (IHC)

Both tumor and adjacent tissue sections (5 *μ*m) were fixed on poly-L-lysine-coated slides. Antibodies were used for immunostaining (*HIF-1α,* 1:100, Abcam;* P4HB*, 1:50, Boster Biological Technology). Biotin-conjugated secondary antibodies were used for visualization. The VECTASTAIN Elite ABC kit (LINARIS, Wertheim, Germany) and diaminobenzidine (DAB) substrate (Boster Biological Technology) were used for immunoperoxidase detection.

### 2.10. Evaluation of IHC Staining

Two experienced pathologists were responsible for evaluating the immunoreactivity of* HIF-1α *and* P4HB. *Staining intensity and the proportion of positive cells were evaluated. The proportions were scored 0 to 4 (negative, positive in ≤10%, positive in >10% and ≤50%, positive in >50% and ≤75%, and positive in >75% of cells). Staining intensity was scored 0 to 3 (negative, weak, moderate, and strong). Two scores were multiplied as the final score (negative, 0; weak, 1-4; moderate, 5-8; strong, 9-12). The proportion was the mean percentage of five areas at 200× magnification.

### 2.11. Lentivirus shRNA, Plasmids, and Transfection

To deplete* HIF-1α*, cells were transfected with shRNA or control vector. Lentiviruses with* HIF-1α *shRNA were obtained from GeneChem (Shanghai, China). After being cultured with puromycin for 72 hours, GC cells were used for screening.* HIF-1α* knockdown was identified and verified by western blotting and RT-PCR. The short-hairpin RNA sequences were as follows: shHIF-1*α*-1: TGTGAGTTCGCATCTTGAT, ctcgag-ATCAAGATGCGAACTCACATT; shHIF-1*α*-2: CTAACTGGACACAGTGTGT, ctcgag-ACACACTGTGTCCAGTTAGTT; shHIF-1*α*-3: GCTGACCAGTTATGATTGT, ctcgag-ACAATCATAACTGGTCAGCTG; the nontargeting control sequence was 5′-TTCTCCGAACGTGTCACGT-3′. Generated by PCR, the full-length human* P4HB* cDNA was subcloned into the pcDNA3.1 vector. Cells were transfected with the HIF-1*α*-shRNA, control-shRNA, pcDNA3.1 vector, pcDNA3.1-*P4HB* plasmid, and HIF-1*α*-shRNA + pcDNA3.1-*P4HB* plasmid in combination or alone using Lipofectamine 2000 (Invitrogen) for 24 h for* in vitro* experiments.

### 2.12. Scrape Motility and Transwell Invasion Assays

The scrape motility assay was used to evaluate cell migration. GC was plated into culture inserts (ibidi, Regensburg, Germany). After incubation for 24 hours, the inserts were removed. An inverted microscope (XDS-100, Shanghai Caikon Optical Instrument Co., Ltd., Shanghai, China) was used to capture the wound monolayers images at 0 and 24 hours after wounding.

The transwell assay was performed to determine cell invasion. Transwell upper chambers coated with gelatin were used to plate GC cells. The lower chambers were coated with 600 *μ*L FBS (30%, Costar, Lowell, MA, USA). Methanol and hematoxylin and eosin were used to fix and stain cells after incubation for 24 hours (Sigma-Aldrich, St. Louis, MO, USA). After removing the upper chambers, the cells on the surface of the lower chambers were migrated and cells were counted and captured by a microscope at 200× magnification in five fields. The average cell number per field represented the migrated cells.

### 2.13. Statistical Analysis

SPSS 19.0 (IBM, Armonk, NY, USA) was used for statistical analysis. The variance between groups used Student's* t*-test; comparisons of multiple groups used one-way analysis of variance (ANOVA).* P*<0.05 was meaningful. All data are presented as mean ± standard deviation.

## 3. Results

### 3.1. Identification of Differentially Expressed Genes

**Figure 1:**
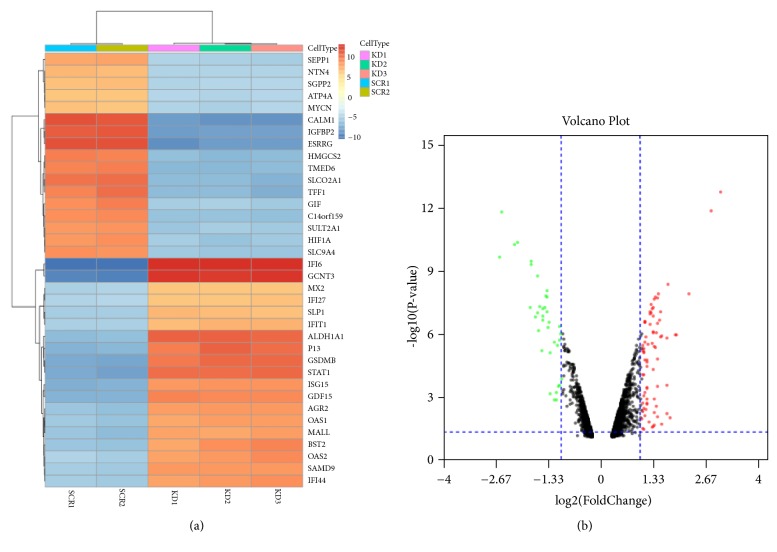
Heatmap and volcano plot exhibited the DEGs. (a) Heatmap of the top 40 DEGs between SCR-AGS and HIF-1*α*-KD-AGS (20 upregulated genes and 20 downregulated genes).* Red: upregulated genes; blue: downregulated genes. *(b) Volcano plot of the differentially expressed mRNAs between SA and paracarcinoma tissues. Red indicates high expression and green indicates low expression (|log⁡2FC| >1 and adjusted* P* value < 0.05). DEGs were calculated by edgeR. 53 high expressed DEGs and 31 low expressed ones. This volcano plot was conducted by the ggplot2 package of R language.

Series matrix files included two SCR-AGS samples and three KD-AGS samples; each chip was analyzed by the edgeR package to identify DEGs. A total of 1,785 DEGs were identified: 886 genes were upregulated in SCR-AGS and 899 genes were upregulated in KD-AGS. A heatmap of the top 20 DEGs in each group is shown in Figure 1(a). Fifty-three upregulated and 31 downregulated DEGs were observed and sorted by fold change (FC ≥ 2,* P*<0.05, Figure 1(b), Supplementary Tables 1 and 2).

### 3.2. Functional Characterization of DEGs

**Figure 2:**
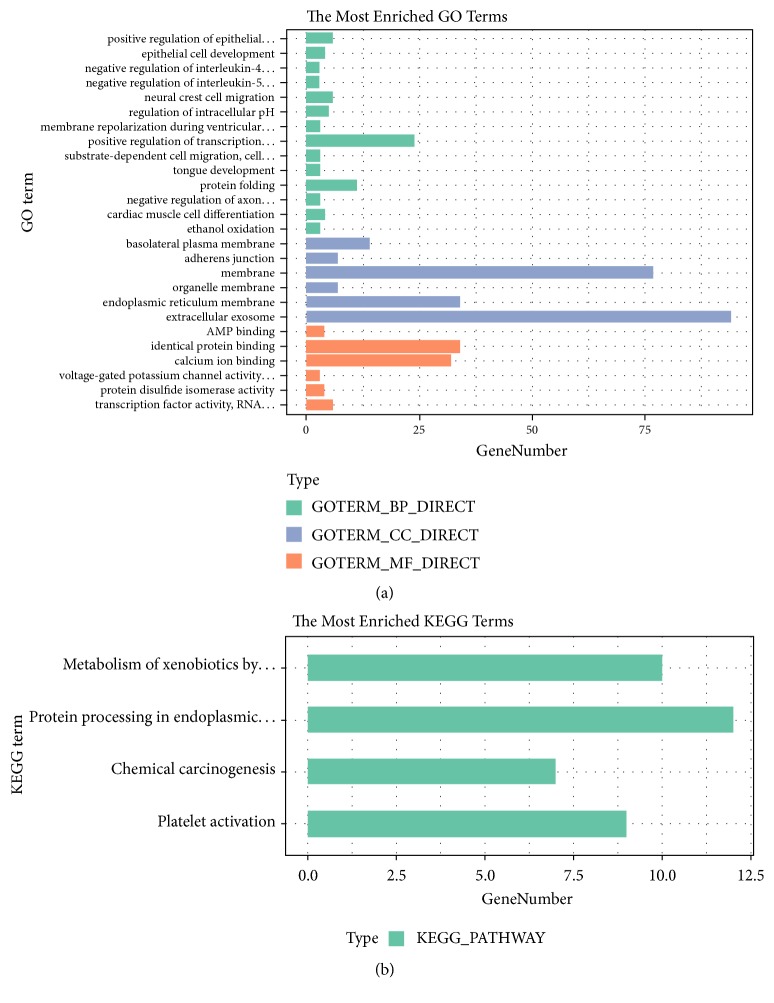
GO functional and KEGG pathway enrichment analyses of DEGs in scrambled AGS cells. (a) GO analysis. (b) KEGG pathway analysis. The* x*-axis shows the number of genes and the* y*-axis shows the GO and KEGG pathway terms.

Upregulated DEGs in SCR-AGS were uploaded to the online database DAVID to identify representative GO terms and KEGG pathways [25] for further elucidation of the functional properties of the DEGs. GO analysis results (Figure 2(a), Supplementary Table 3) showed that upregulated DEGs were significantly enriched in positive regulation of the epithelial-to-mesenchymal transition (EMT) and epithelial cell development and in negative regulation of interleukin-4 and interleukin-5 production under biological process (BP). Under cellular component (CC), the genes were enriched in the basolateral plasma membrane and adherens junction. Additionally, molecular function (MF) analysis revealed genes significantly enriched in AMP binding, identical protein binding, and calcium ion binding.

Figure 2(b) shows the most significantly enriched pathways of the upregulated DEGs according to KEGG pathway analysis. The upregulated DEGs were enriched in metabolism of xenobiotics by cytochrome P450, protein processing in the endoplasmic reticulum, chemical carcinogenesis, and platelet activation (Supplementary Table4).

### 3.3. Module Screening and Centrality Analysis from the PPI Network

**Figure 3:**
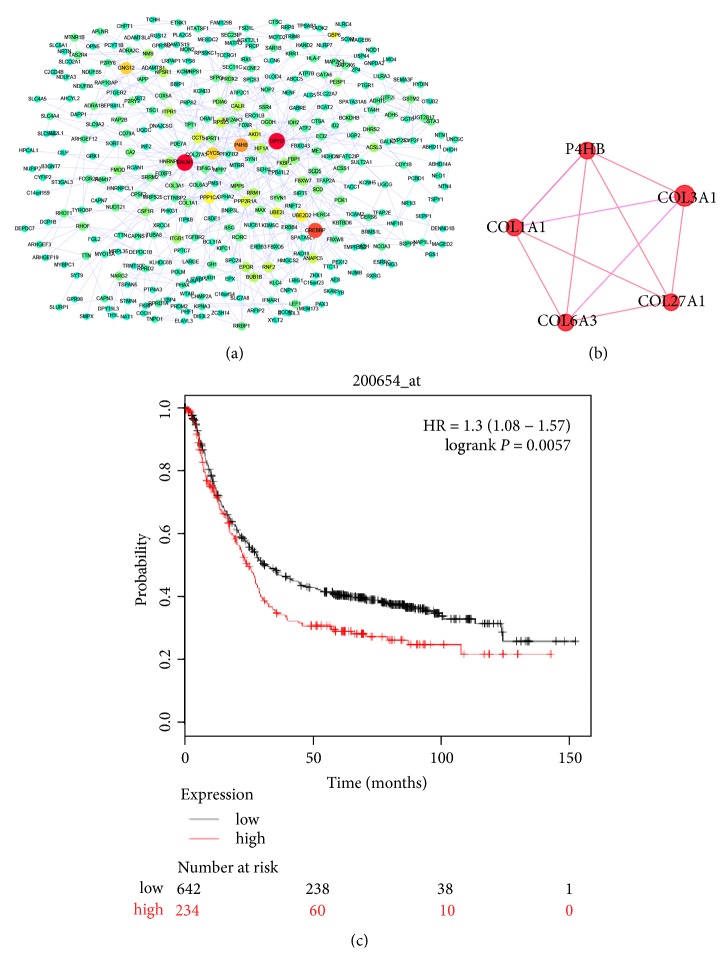
*P4HB* was identified as a target gene of* HIF-1α*. (a) The Protein-protein interaction (PPI) network of the upregulated DEGs in SCR-AGS (edge size was according to the degree, and color was according to the number of directed edges;* red=30, yellow=15, and green=0*). (b) The module contained* P4HB*. (c) The Kaplan-Meier curves of patients with GC based on expression of* P4HB* in the Kaplan-Meier plotter database; patients with low expression of* P4HB* showed a better survival rate (*P* = 0.0057).

PPI network analysis is an important tool for identification of the crucial hub genes in a group of molecules. Using the STRING database, the PPI network for the unregulated genes in SCR was formulated by Cytoscape and is shown in Figure 3(a).

**Figure 4:**
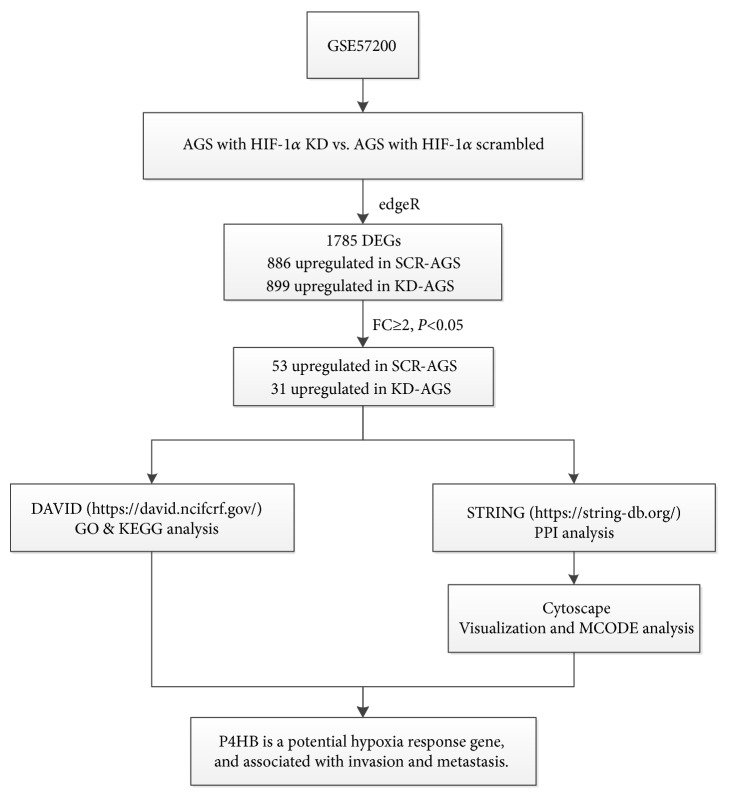
The workflow of bioinformatics analysis.

The possibility of centralities means that a gene is functionally capable of connecting to nodes with other genes in a biological network [26]. Betweenness centrality, closeness centrality, stress centrality, degree centrality, and clustering coefficient are considered the five most important centralities [27]. The first 10 genes of each type were not precisely consistent across the centrality analyses. Therefore, hub genes were those that were shared more than twice between the five types of centralities. Following these criteria, 11 hub genes were obtained from the PPI network: calmodulin 1 (*CALM1*), dihydropyrimidine dehydrogenase (*DPYD*), CREB binding protein (*CREBBP*),* P4HB*, cytochrome c, somatic (*CYCS*),* HIF-1α*, chaperonin containing TCP1 subunit 5 (*CCT5*), protein phosphatase 1 catalytic subunit alpha (*PPP1CA*), protein phosphatase 2 scaffold subunit A alpha (*PPP2R1A*), ribosomal protein S23 (*RPS23*), and phosphorylase kinase catalytic subunit gamma 1 (*PHKG1*). Further, we used the MCODE plug-in to analyze all nodes and edges. The three most important modules were selected (Figure S1). Comprehensive analysis of the hub genes and modules revealed that* P4HB* was the only gene in both areas (centrality and MCODE module) (Figure 3(b)) and has a direct connection with* HIF-1α* (Figure 3(a)). The relationship between the expression of* P4HB* mRNA and clinical outcome was illustrated using the Kaplan-Meier plotter (www.kmplot.com) to show the prognostic value of* P4HB* expression. Further, we plotted survival curves for all patients with GC (Figure 3(c)). High* P4HB* mRNA expression levels were associated with significantly poorer overall survival in all patients with GC (HR 1.3 (1.08-1.57),* P* = 0.0057).  Figure 4 shows the workflow of the bioinformatics analysis.

### 3.4. HIF-1*α* Upregulates P4HB in GC Cells

**Figure 5:**
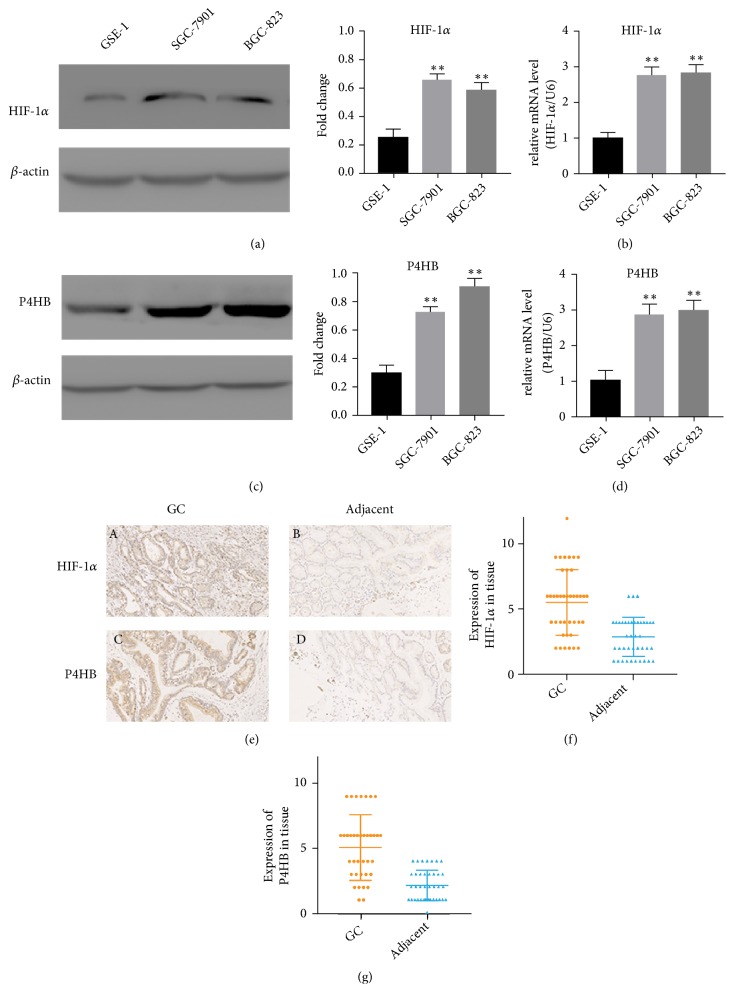
*HIF-1α* and* P4HB* are upregulated in human GC cell lines, and* HIF-1α* regulates the expression of* P4HB* in GC cells. (a, c)* HIF-1α* and* P4HB* protein expression in GC cell lines (SGC-7901 and BGC-823) and the human normal gastric epithelial cell line (GSE-1) by western blot. *β*-actin was used as a loading control; n = 3, *∗∗*:* P *< 0.01. (b, d)* HIF-1α* and* P4HB* mRNA expression in SGC-7901, BGC-823, and GSE-1 by RT-PCR; n = 3, *∗∗*:* P *< 0.01. (e) (A)* HIF-1α* was overexpressed in GC tissue. (B)* HIF-1α* was weakly expressed in adjacent tissues. (C)* P4HB* was overexpressed in GC tissue. (D)* P4HB* was weakly expressed in adjacent tissues. In all figures, 200× magnification was used. (f, g) The score of* HIF-1α* and* P4HB* in 40 GC tissues was of statistical significance compared to that of the corresponding adjacent nontumor specimens by IHC. Data are presented as the mean± SD (n= 40).

Western blot and RT-PCR results showed that both* HIF-1α* and* P4HB *were overexpressed in GC cells compared to their levels in GES-1 cells (Figures 5(a)–5(d),* P *< 0.05) under 1% O_2_. And IHC was used to investigate their expression in 40 GC samples and their noncancerous counterparts.* HIF-1α* was upregulated in GC samples relative to its level in adjacent noncancerous samples (Figures 5(e) and 5(f)). Similar results were found for* P4HB*. Its expression was detected in 39/40 GC samples and there was overexpression in GC tissue compared to levels in adjacent samples (Figures 5(e) and 5(g)).

**Figure 6:**
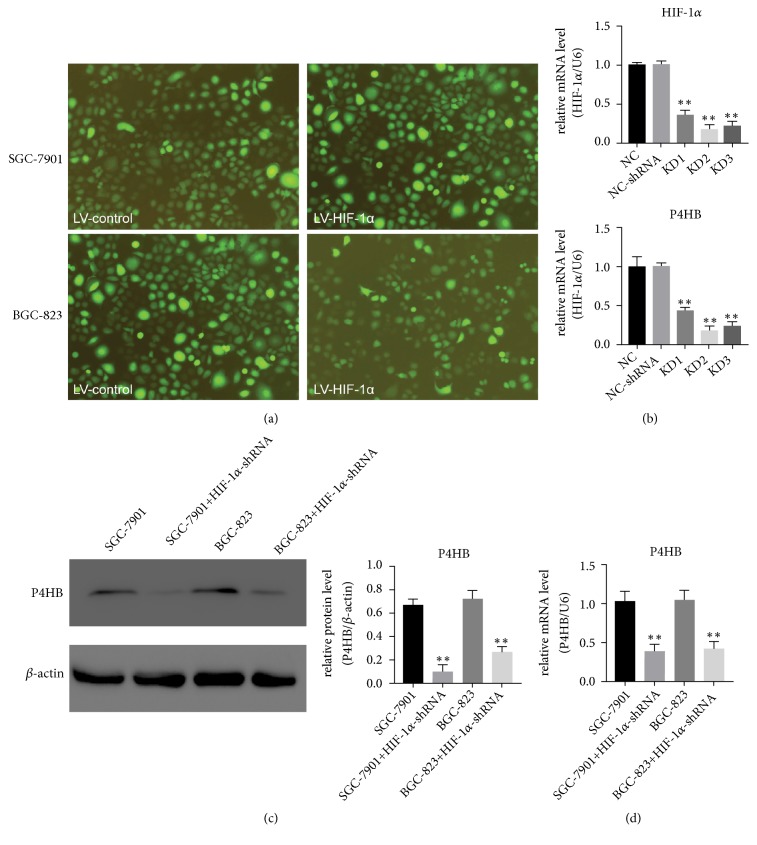
Suppression of* HIF-1α* expression affected* P4HB* expression in GC cells. (a, b) GFP fluorescence and RT-PCR showing the efficiency of* HIF-1α* knockdown compared with that of the scrambled control in GC cells; *∗∗*:* P *< 0.01. (b) GC cells were infected with shRNA-HIF-1*α*-II or LV-control for 24 h.* P4HB* expression levels were determined by western blot and RT-PCR. *∗∗*:* P *< 0.01. (c, d) GC cells were transfected with HIF-1*α* siRNA;* P4HB* protein and mRNA levels were determined by western blot and RT-PCR, respectively.

Highly fluorescent cell bodies visualized by GFP fluorescence indicated lentivirus- (LV-) HIF-1*α* and LV-control (NC) were transfected into GC cells successfully (Figure 6(a)). RT-PCR assays ensured the efficiency of interference and avoided off-target effects. The* HIF-1α* mRNA expression was obviously reduced by shHIF-1*α* compared to the scrambled control. The shRNA-HIF-1*α*-II showed the maximum knockdown efficiency (Figure 6(b),* P *< 0.01). This is consistent with our previous research [28]. With the inhibition of* HIF-1α* by shRNA, the* P4HB* levels were also downregulated (Figures 6(c) and 6(d)).

### 3.5. HIF-1*α* Affected Invasion and Metastasis Mediated by P4HB

**Figure 7:**
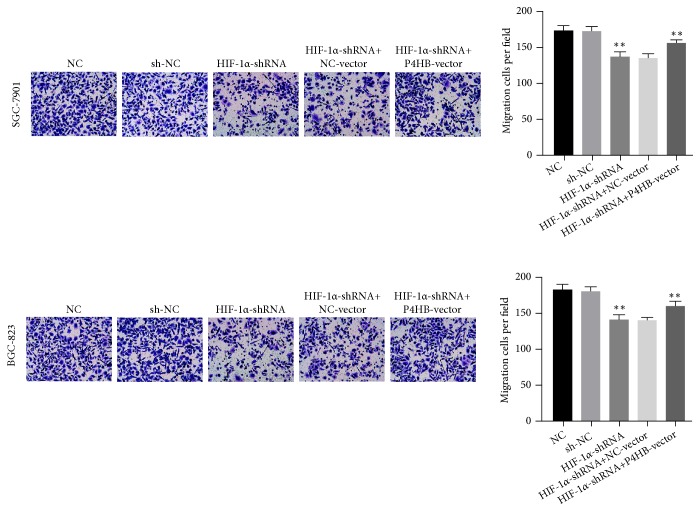
Transwell assays were used to evaluate the role of HIF-1*α* in invasion in HIF-1*α*-knockdown and HIF-1*α*-knockdown + P4HB-overexpression GC cells. In all figures, 200× magnification was used.

**Figure 8:**
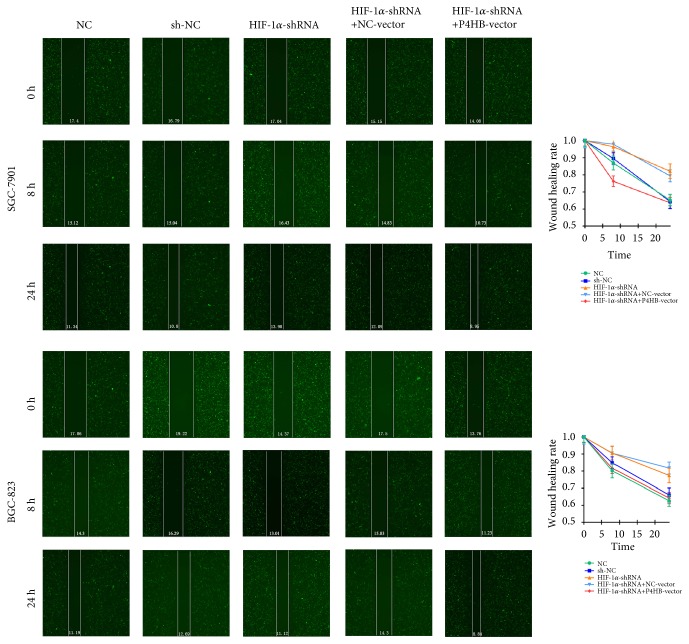
Scrape motility assays were monitored for 24 h in* HIF-1α*-knockdown and* HIF-1α*-knockdown +* P4HB*-overexpression GC cells. In all figures, 200× magnification was used.

In order to study the effect of* HIF-1α* and* P4HB* on the biological function of GC, we knocked down the expression of* HIF-1α* and overexpressed* P4HB*. Transwell experiments and scrape motility assays were performed to test the effect of* HIF-1α* and* P4HB* on cell invasion and metastasis. The results showed that reduction of* HIF-1α* inhibited the invasion and metastasis of GC cells (Figures 7 and 8,* P *< 0.01); this decrease was partially rescued by the overexpression of* P4HB* (Figures 7 and 8,* P *< 0.01). Neither the lentivirus vector nor the pcDNA3.1 plasmid affected* HIF-1α* or* P4HB* expression in GC cells.

## 4. Discussion

With the improvement of NGS, understanding of the molecular pathogenesis of GC has improved, and GC-related molecular mechanisms have been identified [29, 30]. As genes do not function in isolation, they may be divided into “networks” based on their interactions.* HIF-1α* was initially defined as an important regulator of cell adaptation to hypoxia, which plays a crucial role in the tumor microenvironment [31] and is closely linked with invasion and metastasis [32]. Tumor metastasis represents a major challenge to effective cancer treatment. More than 90% of cancer-related deaths are caused by metastasis, and surgery or radiotherapy and/or chemotherapy have only limited effects [33]. Studies have demonstrated that both cells and molecules are regulated by the microenvironment, regardless of whether they are located in primary tumors or distant metastases [34, 35]. Hypoxia activates HIF signaling and influences multiple steps within the metastatic cascade, including invasion, intravasation, and extravasation, and establishment of the premetastatic niche, as well as survival and growth at the distant site [32]. In this study, we extracted raw data from GSE57200 (GPL10558) to identify the DEGs and gene regulatory networks associated with* HIF-1α* in GC. GO terms showed major enrichment of the DEGs involved in the EMT, which is known to be regulated by* HIF-1α *[6]. The results of the centrality and MCODE analyses of PPI showed that* P4HB* may be closely related to* HIF-1α* and represents a potential biomarker of GC invasion and metastasis.

This study used gene ontology and enrichment analyses to identify P4HB as associated with HIF-1*α*, itself indispensable in the hypoxia response and metastasis. We then observed the effects of inhibition and overexpression of HIF-1*α* and P4HB on invasion and metastasis in human gastric cancer cell lines. Our findings demonstrated that P4HB plays a significant role in the regulatory network of HIF-1*α* and is closely linked with invasion and metastasis in gastric cancer cells under hypoxic conditions. We believe that our study makes a significant contribution to the literature because the findings suggest that HIF-1*α* and P4HB may be potential biomarkers of GC.

*P4HB* is the beta subunit of prolyl 4-hydroxylase. Combining with P4HA1 or P4HA2 subunits, they can form a tetrameric enzyme. HIF-1 promotes extracellular matrix remodeling by inducing P4HA1 [36] and P4HA2 in breast cancer [37]. In addition,* P4HA1 *[38] and* P4HA2* [39] are confirmed hypoxia-associated genes and associated with poor prognosis in head and neck squamous cell carcinoma. The expression of P4HA1/P4HA2 affected by hypoxia was also reported in chondrosarcoma cells [40] and soft tissue sarcomas [41]. However, whether HIF-1 directly regulates P4HA1/P4HA2 has not been confirmed.* P4HB* may act as a bridge between HIF-1 and P4HA1/P4HA2.

Although the role of* P4HB* in carcinogenesis remains controversial [42, 43], many studies have demonstrated oncogenic functions for* P4HB *[17–19].* P4HB*, also known as* PDIA1*, belongs to the PDI family, whose role in carcinogenesis has been recently reported. There is abundant evidence supporting the strong association of PDI proteins with a variety of cancers; for example,* P4HB* is a potential target for ovarian cancer therapy [44], and increased PDI activity has been demonstrated in melanoma [45]. The Wnt/*β*-catenin signaling pathway can be activated by* PDIA6*, and the overexpressed* PDIA6* promotes proliferation and growth of bladder cancer cells [46] and HeLa cells [47]. The downregulation of* ERp19* dramatically suppresses cell growth and migration in GC cells [48]. Accordingly, the potential utility of PDI proteins as prognostic factors for clinical use has been suggested. Besides, we have proved the potential value of* P4HB* and* HIF-1α* as prognostic factors for disease-free and overall survival in another study [7]. However, the molecular mechanisms underlying the regulation of* P4HB* in GC and the molecular regulation network associated with* P4HB* are still to be elucidated. In this study, we showed that* HIF-1α* and* P4HB* act as oncogenes* in vitro*, influencing the invasive and metastatic phenotype of GC. Further, our results revealed that* HIF-1α *promotes GC invasion and metastasis by regulating* P4HB*.

Although we reveled and verified the importance of* P4HB* and* HIF-1α* to the invasion and metastasis of GC using comprehensive bioinformatics technology and molecular biological approaches, the present study had some limitations. First, the study lacked any investigation of a normoxic cell culture. Secondly, the expression and function of* P4HB* and* HIF-1α* in GC should be validated in* in vivo* experiments.

In conclusion, we identified hub genes and elucidated the biological and signaling pathways associated with* HIF-1α* in GC cells. Further, we showed that high expression of* P4HB* and* HIF-1α* is correlated with invasion and metastasis in GC. To our knowledge, this is the first study to identify that* P4HB* is a downstream target gene of* HIF-1α*. And its expression is regulated by the latter. The limitations of this study should be addressed in further research.

## Data Availability

The data used to support the findings of this study are included within the article.

## Supplementary Materials

Supplementary Materials Table 1: the upregulated differentially expressed genes in SCR-AGS. Table 2: the upregulated differentially expressed genes in KD-AGS. Table 3: gene ontology analysis of differentially expressed genes in SCR-AGS cells. Table 4: Kyoto Encyclopedia of Genes and Genomes (KEGG) pathway analysis of differentially expressed genes in SCR-AGS cells. Figure S1: the three MCODE modules of hub genes.
