# Supplementary material for: P4HB, a Novel Hypoxia Target Gene Related to Gastric Cancer Invasion and Metastasis
Source: Biomed Res Int. 2019 Jul 30;2019:9749751. doi: 10.1155/2019/9749751 (PMC6699373; doi:10.1155/2019/9749751)
Supplement: Supplementary Materials — Table 1: the upregulated differentially expressed genes in SCR-AGS. Table 2: the upregulated differentially expressed genes in KD-AGS. Table 3: gene ontology analysis of differentially expressed genes in SCR-AGS cells. Table 4: Kyoto Encyclopedia of Genes and Genomes (KEGG) pathway analysis of differentially expressed genes in SCR-AGS cells. Figure S1: the three MCODE modules of hub genes. [file 9749751.f1.zip › Sup Tables and Sup Fig legend_BMRI_2703423.pdf]

Table 1. The up-regulated DEGs in SCR-AGS

Gene	*P*.Value	logFC	Gene title
ALDH1A1	1.51E-13	-2.998	aldehyde dehydrogenase 1 family member A1
ALDH1A1	1.21E-12	-2.758	aldehyde dehydrogenase 1 family member A1
IFI27	1.04E-08	-2.203	interferon alpha inducible protein 27
PI3	9.08E-07	-1.877	peptidase inhibitor 3
OAS2	9.37E-07	-1.851	2'-5'-oligoadenylate synthetase 2
GCNT3	3.63E-09	-1.673	glucosaminyl (N-acetyl) transferase 3, mucin type
GDF15	2.40E-04	-1.649	growth differentiation factor 15
IFI6	1.34E-06	-1.627	interferon alpha inducible protein 6
IFI44	1.11E-06	-1.503	interferon induced protein 44
MALL	7.51E-08	-1.491	mal, T-cell differentiation protein like
IFIT1	1.13E-06	-1.468	interferon induced protein with tetratricopeptide repeats 1
OAS1	1.78E-07	-1.461	2'-5'-oligoadenylate synthetase 1
STAT1	1.01E-08	-1.423	signal transducer and activator of transcription 1
MX2	2.62E-07	-1.411	MX dynamin like GTPase 2
SLPI	1.27E-05	-1.411	secretory leukocyte peptidase inhibitor
SAMD9	1.66E-08	-1.4	sterile alpha motif domain containing 9
BST2	2.60E-04	-1.393	bone marrow stromal cell antigen 2
GSDMB	7.21E-07	-1.359	gasdermin B
ISG15	1.52E-08	-1.353	ISG15 ubiquitin-like modifier
AGR2	3.96E-06	-1.348	anterior gradient 2, protein disulphide isomerase family member
C4BPB	1.97E-08	-1.335	complement component 4 binding protein beta
DDX60	3.38E-06	-1.315	DEXD/H-box helicase 60
EPSTI1	7.48E-07	-1.297	epithelial stromal interaction 1 (breast)
P4HB	4.81E-08	-1.291	prolyl 4-hydroxylase subunit beta
S100A9	4.11E-05	-1.287	S100 calcium binding protein A9
OAS1	1.18E-07	-1.286	2'-5'-oligoadenylate synthetase 1
SIPA1L2	6.79E-07	-1.283	signal induced proliferation associated 1 like 2
GSDMB	2.49E-06	-1.265	gasdermin B
PTPRO	5.47E-08	-1.256	protein tyrosine phosphatase, receptor type O
MUC13	3.24E-08	-1.237	mucin 13, cell surface associated
REG1A	8.08E-08	-1.234	regenerating family member 1 alpha
PTPRO	9.06E-08	-1.219	protein tyrosine phosphatase, receptor type O
IFI6	7.30E-08	-1.218	interferon alpha inducible protein 6
STAT1	3.07E-07	-1.2	signal transducer and activator of transcription 1
IFIT3	2.75E-06	-1.151	interferon induced protein with tetratricopeptide repeats 3
CLDN1	3.05E-05	-1.139	claudin 1
RGS16	3.10E-04	-1.137	regulator of G-protein signaling 16
CEACAM6	1.52E-05	-1.136	carcinoembryonic antigen related cell adhesion molecule 6
PIM1	1.83E-05	-1.131	Pim-1 proto-oncogene, serine/threonine kinase
BST2	3.35E-04	-1.128	bone marrow stromal cell antigen 2
IFIT3	1.18E-06	-1.1	interferon induced protein with tetratricopeptide repeats 3
SYTL2	2.47E-07	-1.091	synaptotagmin like 2

ANXA1	2.14E-07	-1.081	annexin A1
FBXO30	2.33E-07	-1.078	F-box protein 30
TLDC2	2.23E-06	-1.071	TBC/LysM-associated domain containing 2
RASGRP3	1.42E-05	-1.068	RAS guanyl releasing protein 3
EMP1	5.70E-06	-1.051	epithelial membrane protein 1
IRF7	6.32E-06	-1.049	interferon regulatory factor 7
PTRF	7.45E-05	-1.048	polymerase I and transcript release factor
CD68	4.03E-06	-1.047	CD68 molecule
CPS1	3.16E-06	-1.032	carbamoyl-phosphate synthase 1
PCED1B	5.31E-06	-1.027	PC-esterase domain containing 1B
OAS3	7.14E-05	-1.016	2'-5'-oligoadenylate synthetase 3

Table 2. The up-regulated DEGs in KD-AGS

Gene	*P*.Value	logFC	Gene title
FAM46C	1.17E-04	1.036	family with sequence similarity 46 member C
GCNT2	8.72E-07	1.049	glucosaminyl (N-acetyl) transferase 2, I-branching enzyme (I blood group)
PGC	2.42E-04	1.069	progastricsin
ERO1B	1.77E-06	1.071	endoplasmic reticulum oxidoreductase 1 beta
COPS8	3.90E-07	1.075	COP9 signalosome subunit 8
GSTA1	2.70E-04	1.097	glutathione S-transferase alpha 1
NREP	3.09E-06	1.135	neuronal regeneration related protein
CA2	2.16E-06	1.209	carbonic anhydrase 2
CA2	6.44E-06	1.317	carbonic anhydrase 2
NLRP7	2.26E-07	1.356	NLR family pyrin domain containing 7
EFHD2	4.33E-07	1.366	EF-hand domain family member D2
MYCN	7.78E-09	1.391	v-myc avian myelocytomatosis viral oncogene neuroblastoma derived homolog
C14orf159	1.58E-08	1.395	chromosome 14 open reading frame 159
SEPP1	7.82E-08	1.395	selenoprotein P, plasma, 1
TMED6	1.26E-08	1.416	transmembrane p24 trafficking protein 6
SULT2A1	4.60E-08	1.463	sulfotransferase family 2A member 1
CALM1	1.13E-07	1.496	calmodulin 1
HIF1A	1.81E-07	1.498	hypoxia inducible factor 1 alpha subunit
ESRRG	5.15E-08	1.502	estrogen related receptor gamma
TFF1	5.54E-06	1.543	trefoil factor 1
HMGCS2	3.99E-08	1.583	3-hydroxy-3-methylglutaryl-CoA synthase 2
HIF1A	5.98E-07	1.62	hypoxia inducible factor 1 alpha subunit
SLC9A4	1.48E-09	1.627	solute carrier family 9 member A4
ESRRG	8.83E-08	1.627	estrogen related receptor gamma
SGPP2	1.34E-07	1.685	sphingosine-1-phosphate phosphatase 2
NTN4	4.24E-10	1.801	netrin 4
IGFBP2	2.96E-10	1.815	insulin like growth factor binding protein 2
HIF1A	4.59E-08	1.839	hypoxia inducible factor 1 alpha subunit
PUDP	3.67E-11	2.155	pseudouridine 5'-phosphatase
SLCO2A1	4.77E-11	2.215	solute carrier organic anion transporter family member 2A1
ATP4A	1.32E-12	2.564	ATPase H+/K+ transporting alpha subunit
GIF	1.91E-10	2.594	gastric intrinsic factor

Table 3. Gene ontology analysis of differentially expressed genes in scrambled AGS cells.

Category	Term	Count	*P*-Value
GOTERM_BP_DIRECT	positive regulation of epithelial to mesenchymal transition	6	2.40E-03
GOTERM_BP_DIRECT	epithelial cell development	4	3.30E-03
GOTERM_BP_DIRECT	negative regulation of interleukin-4 production	3	7.80E-03
GOTERM_BP_DIRECT	negative regulation of interleukin-5 production	3	7.80E-03
GOTERM_BP_DIRECT	neural crest cell migration	6	9.20E-03
GOTERM_BP_DIRECT	regulation of intracellular pH	5	1.90E-02
GOTERM_BP_DIRECT	membrane repolarization during ventricular cardiac muscle cell action potential	3	2.10E-02
GOTERM_BP_DIRECT	positive regulation of transcription, DNA-templated	24	2.60E-02
GOTERM_BP_DIRECT	substrate-dependent cell migration, cell extension	3	2.60E-02
GOTERM_BP_DIRECT	tongue development	3	3.20E-02
GOTERM_BP_DIRECT	protein folding	11	3.60E-02
GOTERM_BP_DIRECT	negative regulation of axon extension	3	3.80E-02
GOTERM_BP_DIRECT	cardiac muscle cell differentiation	4	4.20E-02
GOTERM_BP_DIRECT	ethanol oxidation	3	4.50E-02
GOTERM_CC_DIRECT	basolateral plasma membrane	14	1.70E-03
GOTERM_CC_DIRECT	adherens junction	7	2.60E-03
GOTERM_CC_DIRECT	membrane	77	3.10E-02
GOTERM_CC_DIRECT	organelle membrane	7	3.50E-02
GOTERM_CC_DIRECT	endoplasmic reticulum membrane	34	4.40E-02
GOTERM_CC_DIRECT	extracellular exosome	94	4.70E-02
GOTERM_MF_DIRECT	AMP binding	4	2.60E-03
GOTERM_MF_DIRECT	identical protein binding	34	1.40E-02
GOTERM_MF_DIRECT	calcium ion binding	32	2.10E-02
GOTERM_MF_DIRECT	voltage-gated potassium channel activity involved in ventricular cardiac muscle cell action potential repolarization	3	2.10E-02
GOTERM_MF_DIRECT	protein disulfide isomerase activity	4	2.60E-02

GOTERM_MF_DIRECT	transcription factor activity, RNA polymerase II distal enhancer sequence-specific binding	6	4.40E-02

Table 4. Kyoto Encyclopedia of Genes and Genomes (KEGG) pathway analysis of differentially expressed genes in scrambled AGS cells.

Category	Term	Count	*P*-Value	Genes
KEGG_PATHWAY	Metabolism of xenobiotics by cytochrome P450	10	4.00E-04	*UGT2B17*, *ADH1B*, *ADH5*, *AKR7A3*, *CYP2F1*, *CYP2S1*, *DHDH*, *GSTA3*, *GSTM2*, *SULT2A1*
KEGG_PATHWAY	Protein processing in endoplasmic reticulum	12	1.50E-02	*DNAJC5G*, *HSPBP1*, *CALR*, *EIF2AK3*, *MAN1B1*, *P4HB*, *PDIA6*, *RRBP1*, *SAR1B*, *SSR4*, *SYVN1*, *UBE2D2*
KEGG_PATHWAY	Chemical carcinogenesis	7	3.60E-02	*NAT1*, *UGT2B17*, *ADH1B*, *ADH5*, *GSTA3*, *GSTM2*, *SULT2A1*
KEGG_PATHWAY	Platelet activation	9	4.60E-02	*FCGR2A*, *ORAI1*, *ARHGEF12*, *COL1A1*, *COL3A1*, *COL27A1*, *ITPR1*, *ITGB1*, *PPP1CA*

Figure S1: The three MCODE modules of hub genes.
