# Supplementary figures and images for: P4HB, a Novel Hypoxia Target Gene Related to Gastric Cancer Invasion and Metastasis
Source: Biomed Res Int. 2019 Jul 30;2019:9749751. doi: 10.1155/2019/9749751 (PMC6699373; doi:10.1155/2019/9749751)

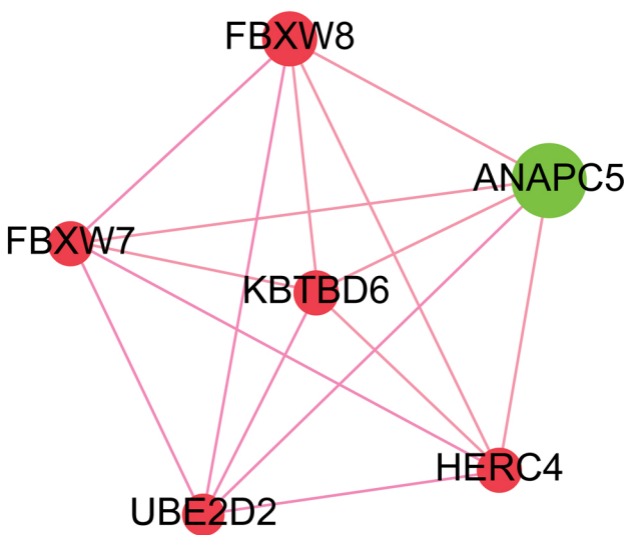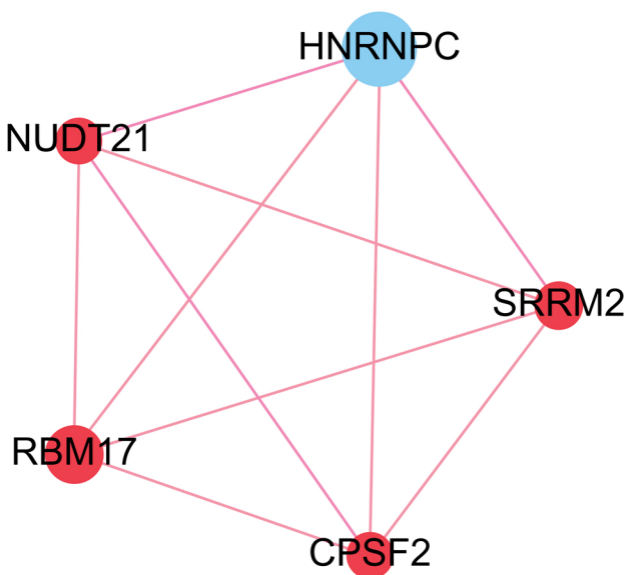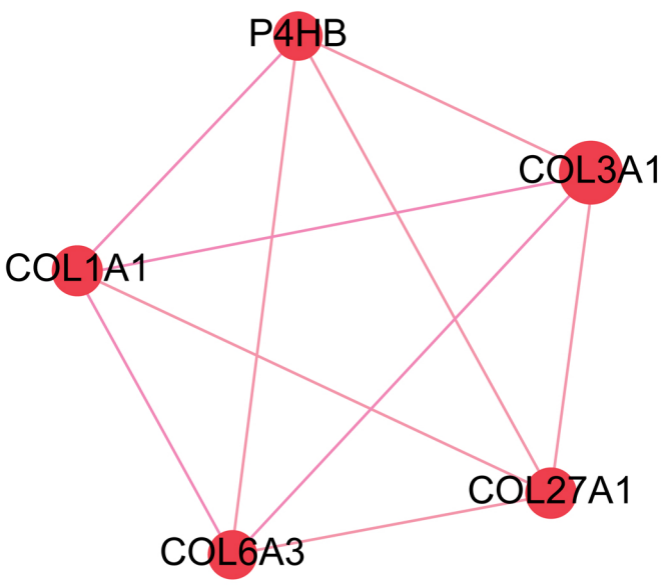

Supplement: Supplementary Materials — Table 1: the upregulated differentially expressed genes in SCR-AGS. Table 2: the upregulated differentially expressed genes in KD-AGS. Table 3: gene ontology analysis of differentially expressed genes in SCR-AGS cells. Table 4: Kyoto Encyclopedia of Genes and Genomes (KEGG) pathway analysis of differentially expressed genes in SCR-AGS cells. Figure S1: the three MCODE modules of hub genes. [file 9749751.f1.zip › Supp Fig_BMRI_2703424.pdf]
